# Applying the Personal and Social Responsibility Model as a School-Wide Project in All Participants: Teachers’ Views

**DOI:** 10.3389/fpsyg.2020.00579

**Published:** 2020-03-27

**Authors:** David Manzano-Sánchez, Luís Conte-Marín, Manuel Gómez-López, Alfonso Valero-Valenzuela

**Affiliations:** Department of Physical Activity and Sport, Faculty of Sports Sciences, University of Murcia, Murcia, Spain

**Keywords:** methodology, innovation, teacher training, education, physical education

## Abstract

The present study aims to apply a program based on Hellison’s Teaching Personal and Social Responsibility Model (TPSR), traditionally used in Physical Education, to other educational participants and examine the assessment of the teachers who carried it out during the 2018–2019 school year. The program was applied over 8 months of one academic year and during at least 60% of the weekly class hours. Initially, 30 teachers participated, of whom 16 were involved in the interviews carried out, all of them being Secondary Education teachers. We used qualitative methodology through content analysis carried out with ATLAS.ti 7.1.3. The instruments used to collect information were semi-structured recorded group interviews. The conclusion is that the Personal and Social Responsibility Model can be applied to all participants in the curriculum and is adaptable to any content and type of student body. As a basis for future research, we suggest the application of the model by all teachers involved in the same school year and the participation of students’ families.

## Introduction

Social demand has led to formal education serving to provide tools for learners to be able to adapt to the constant changes demanded by society. To this end, it is necessary to develop the skills and competences that can help them adapt to these different demands (Larson, 2000). A fundamental aspect of responding to this demand is to promote learner autonomy and self-sufficiency (Oriol et al., 2016), improve the learning environment (Legault and Inzlicht, 2013) and influence learner behavior through Teaching Personal and Social Responsibility Model (TPSR) (Hellison and Wright, 2003) to reduce dependence on teachers’ characteristics in traditional teaching in order to increase cooperative work with classmates (Walkey et al., 2013), in line with the Project Based Learning approach in Stentoft (2016) and Woods (2016) in which learners are significantly involved in their learning by working on joint projects.

In this regard, current education seeks to incorporate new teaching models that allow for a renewal of teaching methodologies that transform the process into meaningful experiences for students in which aspects such as reflection and active participation in classes are especially encouraged (Pérez-López and Rivera-García, 2017). Therefore, it is intended to focus on an approach that allows students to develop their skills and in which they are the real protagonists, rather than teachers’ explicit teaching (Brockbank and McGill, 2002). In other words, teachers become facilitators of learning and students build their own training process (Pérez-López and Rivera-García, 2017).

Motivation is one of the most important variables for implementing an innovative methodology and achieving greater academic adherence and is fundamental to school performance and grades (Shim and Ryan, 2005). The lack of motivational strategies on the part of teachers results in poorer academic performance among students (Tejedor and García-Valcárcel, 2007).

In order to achieve improvements in teaching, new pedagogical models have been developed in recent years (Menéndez and Fernández, 2016), with the so-called TPSR (Hellison, 1978) being highlighted as one of the most appropriate for encouraging students to develop correctly in their social environment, learning to be responsible for themselves and others (Hellison, 1985). This model is currently one of the most powerful tools for the development of values in adolescents (Escartí et al., 2005), its main limitation is the lack of time to apply it, given that it is restricted to a few Physical Education hours a week, the beliefs of students regarding PE, and their difficulties in reflection and dialog (Llopis-Goig et al., 2011).

Furthermore, responsibility has been proven to have positive relationships with motivational constructs, especially with intrinsic motivation (Escartí et al., 2011; Belando et al., 2015; Ortiz et al., 2016), which may indicate that teaching based on the transfer of autonomy and the promotion of responsibility can be appropriate for the improvement of motivation and other social factors along with academic performance (Smithikrai, 2013; Baena-Extremera et al., 2016; Calderón et al., 2016; Granero-Gallegos et al., 2020).

The latest trends in TPSR seek to innovate the model, and thus some studies have hybridized it with Cooperative Learning to find out that TPSR is a very useful model when it comes to meeting the demands of the current educational system and the achievement of competencies through active methodologies (Merino-Barrero et al., 2017). The study by Gordon et al. (2016) implements TPSR with social and emotional learning (SEL) (Gould and Carson, 2008) in line with TPSR approaches and strategies and achieves very interesting results in student self-control. Furthermore, the study by Alcalá et al. (2019), in which they applied TPSR with Pre-school, Primary and Physical Education university students in Spain, Costa Rica and Chile is also worth highlighting. The participants’ perception of the pedagogical possibilities was very positive with regard to their future as teachers, and they agreed on the capacity to promote their professional development in the study by Llopis-Goig et al. (2011).

Thus, teacher training is considered a dimension of capital importance because of its decisive impact on the quality of educational inclusion (León Guerrero, 2011). In this context, the professional development of teachers should be understood as any attempt to improve educational practice, beliefs and professional and personal knowledge with the aim of increasing the quality of teaching, research and management, individually and together with colleagues, in order to improve student learning (Imbernón, 2013). However, professional development is more than just training; it is the product of the pedagogical development that teachers acquire throughout their lives, their knowledge and understanding of themselves as teachers, the cognitive and emotional development that they carry out individually and with their colleagues at school, and the theoretical development of educational matters (Pegalajar-Palomino, 2014).

This work was preceded by one (Manzano and Valero-Valenzuela, 2019) in which a pilot study was carried out applying TPSR in a primary school, and which obtained improvements in students’ self-concept, responsibility, motivation and autonomy, in addition to high levels of satisfaction with the methodology on the part of the students and teachers involved. In addition to this, there is study (Manzano-Sánchez and Valero-Valenzuela, 2019), in which more than 300 primary and secondary school students obtained improvements in responsibility, intrinsic motivation, basic psychological needs, classroom climate and pro-social behavior by applying the model and in which a series of teacher interviews were conducted with a very positive evaluation of the model by the students.

The main objective of the present study is to apply the TPSR in the general educational context, to examine the perception of the teachers involved in terms of its applicability, future perspectives and positive and negative aspects of the experience.

## Materials and Methods

### Design

A qualitative research study was carried out by using a descriptive and cross-sectional method with semi-structured interviews with teachers participating in the implementation of the TPSR, which were carried out once the model application was completed and with sustained training and assessment for teachers (Montero and León, 2007).

### Participants

Participants were selected according to accessibility and convenience. They included 30 Secondary Education teachers from public schools using the Personal and Social Responsibility Model. The teachers who participated in the interviews were a total of 16. None of the participants in the interviews had previous experience with the TPSR. The teachers were recorded in video and their behavior was analyzed by experts qualified in observational methodology and the TPSR.

### Instruments

At the same time, a qualitative analysis of teachers was carried out at the end of the process through semi-structured interviews in order to obtain an internal perspective of their experience (Patton, 2002) in their own school, with 13 questions mainly coming from the study Sánchez-Alcaraz et al. (2019): (1) Do you feel that you have more tools available to teach in schools and deal with children with coexistence issues? (2) Do you feel that you are sufficiently trained to implement the TPSR? (3) What are the main problems that have arisen? (4) How do you feel when you apply the TPSR? (5) What are the most innovative aspects that you feel the TPSR is bringing to your classes? (6) What do you think can be improved in the application of the TPSR? (7) What characteristics of the students do you think could be more adequate for an appropriate application of the TPSR? Do you think teachers’ previous experience is important? (8) What improvements do you think could be made to the contents of the TPSR? (9) Do you think that the TPSR actually works in terms of the inclusion of values, attitudes and socially adequate values? (10) Do you think that through the TPSR students learn the contents of the subject as well as attitudes and values? (11) What advantages have you found in the TPSR with respect to the methodology you have used until now? (12) Are the tasks better adapted to the interests of the students? (13) Is there anything else you would like to add?

### Procedure

We included Informed consent (confidential treatment of data, participation in the study and filming of sessions) for parents and students and a letter of presentation was sent to schools along with the report of the Ethics Committee of the University of Murcia (1685/2017). The program covered 8 months. The contents were selected in accordance with current educational laws (State Agency State Gazette, 2013).

### Intervention Program

The sessions featured a modification of Hellison’s session format (Hellison, 2011) given that they included four parts rather than five: (1) initial greeting: the teacher interacted with students to create links with them, (2) awareness talk: the teacher presented the objectives of the session in academic and values terms according to the level of the responsibility model, (3) activity plan, where most of the practical class was carried out, integrating responsibility strategies in the various tasks, and (4) group meeting and self-evaluation: at the end of each session, the teacher and students shared their perceptions regarding responsibility and individual behaviors, the class and the teacher’s performance by raising their thumb upward (positive evaluation), to the side (middle) or downward (negative evaluation). Teachers used both general strategies to implement the TPSR (e.g., assigning tasks, providing opportunities for success, defining roles) and specific strategies (e.g., reciprocal teaching, cooperative groups, personal work plan). Similarly, these strategies were also used to resolve individual (e.g., 5 Clean Days) and collective (e.g., Grandma’s Law) conflicts, fully integrating the TPSR into all classes in addition to Physical Education (Escartí et al., 2013).

### Teacher Training

The implementation of any educational program requires specific teacher training (Li et al., 2008). Teachers were trained in the TPSR in a two-phase approach: (1) A 5 h course on the model’s theory and practice in which they were explained how to design classroom climates according to the model and were provided with comprehensive and specific strategies for accountability development, as well as a “model guide” so that they could review the various strategies undertaken as well as others. (2) Continuous training: during the implementation of the program (8 months) the principal researcher met with the teachers on several occasions and through different channels. In the first week, the teachers had to hand in a document reflecting the structure of a session of their own class applying the model at the first level, with their corresponding activities and strategies, with the principal researcher providing feedback to the teachers as appropriate. In the second week, the sessions were implemented in the different participants, and a minimum of one session per month was filmed and evaluated by the research team. In the following week, a report was given with the session and the aspects to be modified, and this sequencing was carried out throughout the intervention, as well as a quarterly meeting to discuss the intervention among the teachers. The students learned responsibility progressively, moving through the different levels (Escartí et al., 2013). However, level 5 was involved from the beginning, in an attempt to transfer it to students’ lives.

### Interview Preparation

To draw up the interview questions, the aspects covered in the TPSR were taken into account, as well as those that could be of interest as an object of the investigation. For this purpose, several questions were selected and analyzed by various researchers and/or teachers who had applied the TPSR, ending with the selection of the 13 questions above.

The interviews were carried out dividing the teachers into two groups of seven and nine people who were interviewed by two researchers who only intervened during the interview to formulate the questions in order to avoid possible biases. The duration of the interviews was 75 min and the answers were recorded through a wireless microphone in order to analyze the results later.

### Data Analysis

The results were analyzed using ATLAS V. 7.1.3, powerful software for the qualitative analysis of large bodies of textual, graphical, audio and video data (Morales-Sánchez et al., 2014).

To analyze the qualitative content of the interviews, the video recordings of the interviews (primary documents) were incorporated for analysis in multimedia format, having been organized and stored in a single file (Hermeneutic Unit). This file contains all the information produced in the course of the analysis in addition to the primary documents and contains codes, notes and memos, quotations and network views.

**FIGURE 1:**
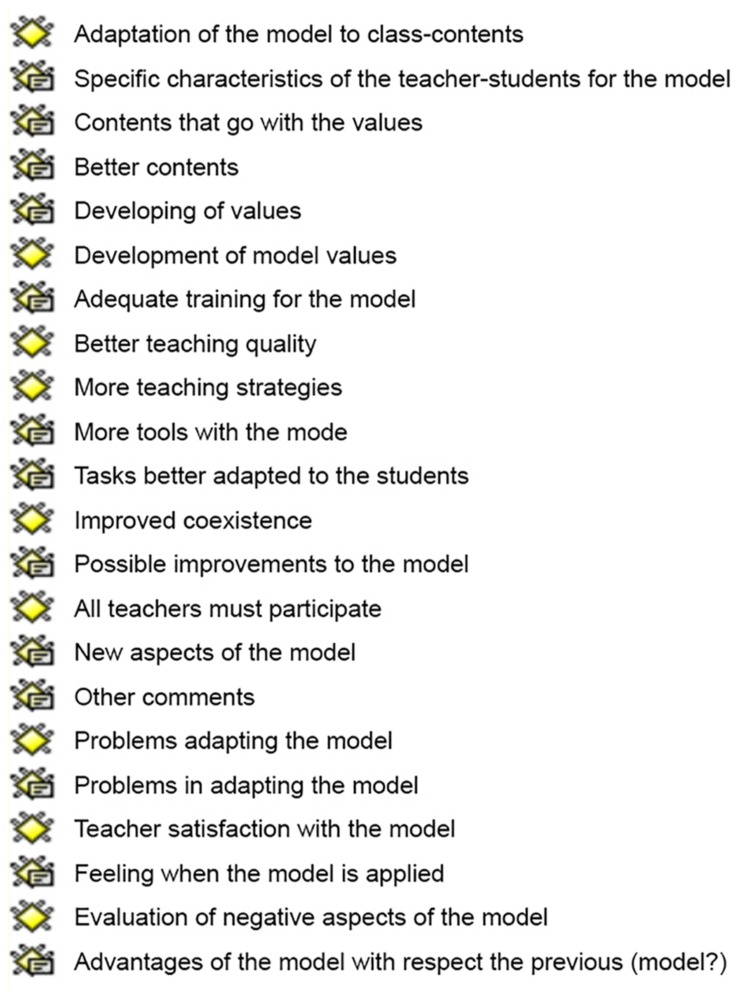
Code families and new codes.

Each of the primary documents corresponded to one of the participants and the answers they gave. It should be noted that the answers were recorded literally and that some participants did not contribute to all the answers by agreeing with the rest of the participants or not answering at all. The quotes are each of the phrases or sentences that have been included in the study. The notes or memos correspond to segments of the recordings that were noteworthy from the primary documents and that are associated with different codes. The codes were the different aspects of the main analysis, reflected in 13 families of codes corresponding to each of the questions and eight new codes referring to the analyzed variables that emerged from the previous ones that were the object of interest (Figure 1). Finally, the network views reflect the connection between the different codes.

## Results

**FIGURE 2:**
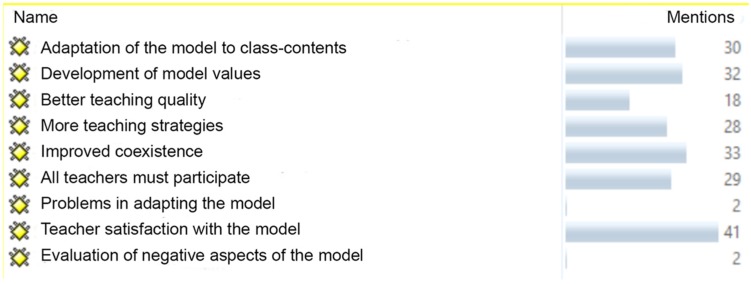
Justification for the codes.

The analysis of the content of the interviews was carried by reducing the different citations in the different codes elaborated according to the objectives of the present research regarding the characteristics of the TPSR and the contributions for future research and aspects to be highlighted and improved (Figure 2).

The “Teacher Satisfaction with the Model” code had a frequency of 41 and was the one most reflected in the interviews. In this regard, it is worth noting some statements in relation to satisfaction such as, “Thanks to the model, I feel useful, I mean what we said before, I see that I am spending my energy or investing it in a productive way”. Also noteworthy is the contribution in one of the interviews by a teacher who said that, “The Responsibility Model is the best-kept secret”.

Worthy of further note are those least related to “Improving Coexistence”, “Promoting Model Values” and “Positive Adaptation of the Model to the Class-Content”. In this regard, it has been a very positive contribution to have a high level of rationale for this last code, given that one of the main objectives of the application of the TPSR to all participants is to determine its capacity to adapt to these and the different students, with some comments such as “I have not had to modify any content, nothing different, so really totally adapted to them and giving the contents” (sic) or others such as “It is valid for any course, it is necessary for motivation and attention and these are the two variables that we have to promote as much as possible”.

On the other side of the coin is the fact that the negative aspects had a very low number of mentions. Specifically, the “Negative Assessment of Model Aspects” occurred on two occasions, specifically in reference to the systematic nature of the application and the importance of leaving time for the final part of the session, for example, “You have to be careful to really comply systematically with the structure of the session or it will remain that way, simply strategies that you apply as you can, you must be systematic”. As for the “Model Adaptation Problems”, only two results were found, with reflections related to the Second year of High School, “I have had problems with Second year of High School, they have difficulties in many areas. We have taught these students since they were little…and we always complain that they are good but very talkative”.

Finally, another of the objectives was to see the future perspectives of the model and suggestions; we created a new Code called “Need for participation of all teachers” which had 29 mentions, 26 of these referring to teachers and three to the importance of including families. By way of example, the following reflection, “It should be a uniform thing and if possible, unify reward, levels…something essential, that they always know that from the beginning of the course they have an ID card that works for all the teachers and all the participants”.

**FIGURE 3:**
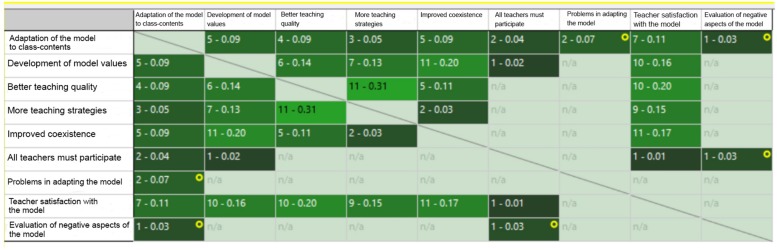
Code co-ocurrence table.

A co-occurrence analysis was also carried out, the relationships between codes with similar dimensions being reflected in Table format (Figure 3). The maximum co-occurrence was between “higher quality of teaching” and “More Strategies in teaching” with a frequency of 11 and an index of 0.31. Secondly, the co-occurrence between “Promotion of model values” and “Improvement of coexistence” with a frequency of 11 and an index of 0.20 and “Higher quality of teaching” with “Teacher satisfaction with the model”, with a frequency of 10 and an index of 0.20 is worth noting.

**FIGURE 4:**
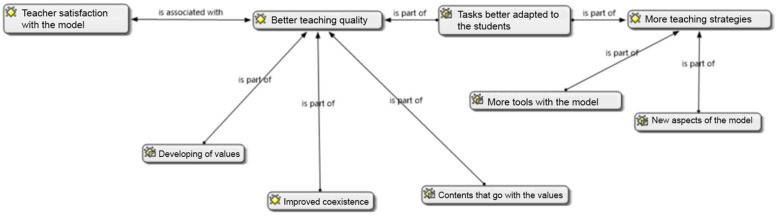
Co-occurence network.

Finally, it is important to note a network where all the co-occurrences were imported (Figure 4). Figure 4 shows that the highest quality of teaching is associated with teacher satisfaction with the model. In this regard, various codes were part of the highest quality of teaching: the promotion of values, the improvement of coexistence, the greater adaptation of tasks, and the ease of providing content as well as the values of the model.

## Discussion

The main objective of the present study was to apply the TPSR in the general educational context to measure the perception of the teachers involved in terms of its application, future perspectives and positive and negative aspects of the experience.

In this regard, it should be noted that of all the codes generated, the one that had the greatest occurrence in the interviews was “satisfaction with the model”. Furthermore, some of the interviews noted the importance of continuing to apply the model for a long time to improve performance and that it should last throughout the school year, in line with the study by Llopis-Goig et al. (2011) which indicates that improvements in the model in terms of responsibility require a long duration, as also indicated by Manzano-Sánchez et al. (2019) where, with an application period of 4 months, improvements were obtained in only some of the variables, its duration being a limitation. Similar is the case of Sánchez-Alcaraz et al. (2019) where the importance of applying the model throughout the school year and in all participants is highlighted in the interviews carried out. In addition to this, it should be noted that Llopis-Goig et al. (2011) have pointed out the importance of involving the entire educational community and the family environment, the latter also being reflected by the teachers participating in this study.

The TPSR was also perceived by the teachers as applicable to all contents and participants, with comments such as “All contents are adapted to one or other levels always, but the question is group level and how to adapt it to this”, which is a positive assessment as to the capacity of the TPSR to adapt to the contents while working on educational values.” You are no longer limited only to working on the contents of the subject, but you already have a system to measure, evaluate and work on other aspects related to behavior and conduct”. All of this is in line with the study by Manzano and Valero-Valenzuela (2019), here the teacher who participated in the study indicated that he was able to adapt the model to all the areas he taught in his group in Primary Education.

It is worth noting that the teachers’ perception was that TPSR can be suitable not only for difficult students but for any type of student. Furthermore, certain interviews reflected the importance of teachers participating in the methodology, and that this should increase the time students are taught through the model, “The time of exposure that the student has to the model has an impact and is important, of course”. This does not coincide with the study by Sánchez-Alcaraz et al. (2013), in which teachers felt that content, student body and teacher experience are determining factors in the application of TPSR.

With regard to the classroom climate, very positive results were also obtained, with the code “improvement of coexistence” having a total of 33 mentions that represented the second-highest value behind satisfaction, with some comments such as that of a teacher with a difficult group, “With the model now they respect each other and these things, now they let others work and this is already an achievement in itself, it means reaching and surpassing level 1 and that is a lot”, thus supporting the study by Caballero (2015) where in applying the TPSR the participants obtained improvements in students’ classroom climate with activities in the natural environment. Several teachers referred to aspects related to better student behavior, following the line of the aforementioned author and Carreres (2014) where the TPSR improved not only personal and social responsibility but also pro-social behavior. The study by Escartì et al. (2010) confirms that it helped teachers to structure classes and promoted the learning of responsible student behaviors. A significant increase in self-regulatory and self-efficacy was also observed in the participants of the intervention group.

The only study that has applied the TPSR in secondary schools and that has carried out interviews with teachers found much in line with those of this intervention. Thus, Manzano-Sánchez and Valero-Valenzuela (2019), showed high satisfaction with the model in the interviews conducted, a positive evaluation in terms of its possibilities for promoting values and wanting to continue its application in future courses, aspects also reflected within the different reflections on this study by the teachers. We therefore do not agree with the study by García et al. (2014), where the teaching staff indicate that the innovative methodologies for promoting autonomy and responsibility are perceived as complex to apply due to the limited time available, given that there was only one comment that indicated “the day I get confused I don’t get to the evaluation, or I set the alarm on my mobile phone 5 min before the end of the class”. Furthermore, the possibility of hybridizing with other pedagogical models such as Cooperative Learning, could improve some of the potential or educational results that TPSR has in a unique way (González-Víllora et al., 2019).

It should be noted that the novelty of this study in terms of its scope (educational context in general) may serve as a reference point for future work in education, since although very good results have been obtained in terms of teacher assessment, they could only be contrasted with studies in Physical Education, extracurricular sports activities or other sports activities, with the exception of the studies by Manzano and Valero-Valenzuela (2019) and Manzano-Sánchez and Valero-Valenzuela (2019). Finally, the use of the mixed methods methodology helps to draw conclusions taking into account different points of view and perspectives.

## Conclusion

The TPSR can be applied to all participants in the curriculum and is perceived as appropriate for teachers. Its applicability does not have more suitable content or more specific groups than others, but it can be used for any teacher or course independently of these aspects. It was perceived as appropriate to improve educational values without detriment of the contents, indicating even more simplicity to teach contents and a new motivation for teachers. It is suggested that the application of the TPSR should be carried out by all teachers participating with the same class group in order to improve the benefits and involve families in the process. Future lines of research could consider the possibility of replicating this study in other contexts within the educational system (for instance elementary or primary education), using a methodology similar to that of this study, combining quantitative and qualitative instruments.

## Data Availability Statement

The raw data supporting the conclusions of this article will be made available by the authors, without undue reservation, to any qualified researcher.

## Ethics Statement

The studies involving human participants were reviewed and approved by the Ethics Committee of the University of Murcia REF-1685/2017. Written informed consent to participate in this study was provided by the participants, participants’ legal guardian/next of kin.

## Author Contributions

DM-S and AV-V developed the methodological proposal and data analysis. MG-L described the conclusions and references. DM-S realized the literature review and wrote the part of the theoretical frame. LC-M collaborated in data analysis and redaction of results.

## Conflict of Interest

The authors declare that the research was conducted in the absence of any commercial or financial relationships that could be construed as a potential conflict of interest.
